# A Non-Operative Approach to Rectal Cancer after Chemo-Radiotherapy: Case Series and Review

**Published:** 2019-02-26

**Authors:** Abeer Arain, Priyank Patel

**Affiliations:** Carle Foundation Hospital, Urbana, IL

**Keywords:** rectal cancer, treatment, surgery, chemoradiation, wait and watch

## Abstract

**Introduction:**

Chemotherapy administered concurrently with radiotherapy for locally-advanced rectal cancer prior to surgery is a standard of care approach. A fraction of patients after chemo-radiotherapy achieve pathological complete remission. Our aim was to evaluate patients treated only with a non-operative approach of only chemo-radiotherapy followed by observation at a community cancer center.

**Methods:**

Medical charts of the patients who were treated for locally advanced rectal cancer and treated with chemo-radiation therapy alone from January 1, 2000 through May 1, 2017 at a Midwestern cancer center were reviewed. The clinical course of the patients was followed from the time of the cancer diagnosis through their last available clinical record.

**Results:**

A series of three cases were reviewed with locally-advanced distal rectal cancers treated with a non-operative approach.

**Conclusions:**

Watchful waiting for patients with locally advanced distal rectal cancer who have complete clinical response with neoadjuvant chemotherapy and radiation might be an effective treatment strategy.

## INTRODUCTION

Post-operative morbidity secondary to total mesorectal excision in rectal cancer patients has increased the interest in organ preserving strategies significantly.^1^ Alternative treatment strategies without radical surgery not only reduces the post-operative morbidity, but also lowers the need for intestinal stomas and functional disorders of the anorectal tract.^2^ Several European trials have documented watch-and-wait approaches that include treatment with chemo-radiation followed by regular surveillance and monitoring for tumor recurrence.^3^,^4^ The complete response rate with watch-and-wait approaches has ranged from 5 to 60%.^3^,^4^ This variation may be due to different follow-up durations and surveillance intensity.

A study by Habr-Gama et al.^5^ reported local recurrence in 31% of patients with initial regrowth and late recurrences. More than half of the recurrences developed in the first 12 months. The authors described that “salvage therapy is possible in > 90% of recurrences, leading to 94% local disease control and 78% organ preservation”. The same authors conducted a prospective study on 70 patients with T2–T4, N0–2, M0, neoadjuvant chemo-radiotherapy that included 54 Gy and 5-FU/leucovorin delivered in six cycles.^6^ Patients in this study were assessed for tumor response at 10 weeks from radiation therapy. About 47 patients had initial complete response and about eight patients developed regrowth in the first 12 months of follow-up.

The aim of this case-series was to review and report the number of cases treated for T2–T4, N0–N2, and M0 rectal cancer with a non-surgical approach and to evaluate the clinical outcome. Our goal was to augment the findings from the existing literature on the watch-and-wait management of locally advanced distal rectal cancer patients.

## METHODS

This study was a case series consisting of three patients. All of the patients included in the study had rectal cancer treated with chemotherapy and radiation with no surgical intervention. The patients were identified using cancer registry databases and the clinical course was retrospectively followed from the time of their histologically proven malignancy until their last available clinical record.

Medical charts of the patients who were treated for locally advanced rectal cancer and treated with chemo-radiation therapy alone from January 1, 2000 through May 1, 2017 at Our Community Cancer Center at Carle Foundation Hospital were reviewed. Seventeen patients were identified, but three presented without metastatic disease and were included in the case series. Patient inclusion required a diagnoses of rectal cancer, treated only with chemotherapy and radiation and no surgical intervention. All or part of the cancer treatment was received at the Carle Foundation Hospital. Patients who did not meet the above criteria were excluded from the case-series.

The clinical course of the patients was followed from the time of the cancer diagnosis through their last available clinical record (Table 1). Approval from the institutional review board committee of Carle Foundation Hospital was obtained prior to the study.

## RESULTS

### Case 1

A 92-year-old female presented to her primary care physician with rectal bleeding. A 3 cm mass at the distal rectum was found on colonoscopy. Needle biopsy of the mass revealed moderately differentiated rectal carcinoma at the lower rectal valve. Baseline computed tomography (CT) diagnostic imaging of the chest, abdomen, and pelvis indicated wall thickening of the distal rectum with no significant pelvic adenopathy and no overt findings for metastatic disease. The patient had T3N0M0 adenocarcinoma of the distal rectum by transrectal ultrasound. Based on her poor cardiac health due to a recent myocardial infarction and advanced age, the patient was regarded as high-risk for the hemicolectomy. She was started on capecitabine chemotherapy along with radiation therapy. She completed 25 fractions of radiation therapy. A total of 50 Gy of radiation treatment was administered to her rectum. The patient tolerated the treatment well with improvement in her rectal bleeding and gradual recovery to baseline. The patient continued to be in remission at 24 months post-treatment without evidence of metastasis on surveillance scans.

### Case 2

An 84-year-old male was found to have adenocarcinoma of the distal rectum after he presented with a complaint of pressure and discomfort with bowel movements along with intermittent rectal bleeding. He had a 3 cm exophytic mass in the rectum and the biopsy reported moderately differentiated adenocarcinoma of the rectum invading submucosa and muscularis propria. CT scan did not reveal distant metastasis. Clinical staging with the aid of transrectal ultrasound was determined to be cT3N1. Due to the patient’s personal preference, he refused surgery and wanted only non-surgical options. The patient was given capecitabine along with radiation therapy of 50 Gy in 25 fractions. The patient did not have evidence of local or distant failure on clinical exams and surveillance studies after 36 months post-treatment.

### Case 3

A 65-year-old male with no significant past medical history and no family history of colorectal cancer presented to his primary care provider with a complaint of intermittent rectal bleeding and alteration in bowel habits. He underwent colonoscopy and had a 3 cm mid-rectal neoplasm which was hemi-circumferential, lobulated, ulcerated, and adjacent to the 2nd rectal valve. Biopsy demonstrated adenocarcinoma arising in the background of an adenomatous polyp. Staging CT scan of the chest, abdomen, and pelvis was unremarkable for distant metastasis. The tumor was staged as T3N1M0 by transrectal ultrasound. Due to this patient’s personal preference, he was treated non-operatively. He received chemotherapy with 5-fluorouracil and leucovorin concurrently with radiation therapy. Thirty months post-treatment, he was without evidence of recurrence.

## DISCUSSION

Treatment options for patients with T2–T4 colorectal cancer that lead to complete clinical response after neoadjuvant chemo-radiotherapy remain controversial.^2^ The most common type of treatment is neoadjuvant chemotherapy and radiation followed by surgical management.^7^ The addition of chemotherapy to the neoadjuvant radiation has been found to improve localized disease control. Combination neoadjuvant treatment results in tumor downstaging, peri-rectal node sterilization, and preparation for surgical treatment. However, the absence of residual tumor cells in the resected surgical specimens after neoadjuvant chemoradiotherapy raised the issue of whether surgical treatment is necessary. This gave rise to a new treatment approach in patients with locally advanced rectal cancer, in which the patient is monitored carefully for any tumor recurrence after the chemo-radiation therapy. This is referred to as the strict surveillance method. This organ preserving strategy was designed to avoid major surgery which typically is associated with significant postoperative morbidity, functional disorders such as urinary, sexual, and anorectal problems, and the need for intestinal stomas.^2^ Tumors that recur after the non-surgical approach frequently are amenable to surgical resection.^5^

After a long debate for several years, the wait-and-watch approach for locally advanced rectal cancer is more well-known in the literature.^5^ Trials in Europe and United States have supported the management of rectal cancer patients staged at T2–T3 with close surveillance methods using the wait-and-watch approach after receiving neoadjuvant chemoradiation therapy.^5^,^8^,^9^ These studies demonstrated radiological findings showing complete clinical or pathological response using modalities such as MRI or PET CT scans.

A study conducted by Habr-Gama et al.^2^ looked at the wait-and-watch approach. In the study, patients with distal rectal cancer who achieved a complete clinical response with neoadjuvant chemotherapy and radiation were followed without undergoing any surgical management. The patients were followed for approximately 60 months. The results were compared with the control group who underwent total mesorectal excision (TME). The patients in the wait-and-watch category showed impressive results with five-year overall survival of 93% and disease-free survival of 85%.^1^,^2^

The wait-and-watch approach has been used with selected patients at our cancer center, specifically for patients with locally advanced rectal cancer. The three patients in our case-series have shown optimistic outcomes with no tumor recurrence, at least in the first 24 months after neoadjuvant chemoradiation therapy without any surgical intervention. Our study supported the findings of Maas et al.^4^ who conducted a prospective study on their patients using the wait-and-watch approach. Their study included 21 patients with locally advanced rectal cancer with a complete clinical response after chemo-radiotherapy. Patients underwent surveillance follow-ups every three to six months using MRI, endoscopy, or CT scans. Mean follow-up was about 25 months. Only one patient developed local recurrence while the other 20 patients were alive without any signs of tumor recurrence. The two-year disease free survival rate for these patients was 89% (95% CI, 43% – 98%), and the cumulative probability for two year overall survival was 100%. In the control group, which included the patients who underwent surgery after the neoadjuvant chemo-radiotherapy, the two-year disease free survival was about 93% with an overall survival of 91% respectively. The three patients in our study also showed average overall disease free survival of 24 months with a two-year overall survival of 100%.

Renehan et al.^10^ conducted a study in the UK of 357 patients with locally advanced rectal cancer;228 of them underwent surgical treatment after neoadjuvant, and the remaining 129 patients stayed on the wait-and-watch approach. Of these 129 patients, 44 had local tumor regrowth and 36 patients out of 41 received a salvage therapy. In the matched analyses, the study did not find any difference in the three year overall survival (96% in the surgical vs 87% in the wait-and-watch group). Patients managed with wait-and-watch had significantly better three-year colostomy free survival than those who underwent surgical resection (74% vs 47%).

To avoid the extensive procedure of abdominoperineal resection, Appelt et al.^11^ studied high dose radiation and chemotherapy in T2–T3, N0–N1 distal rectal adenocarcinomas. About 60 Gy in 30 fractions to tumor and 50 Gy in 30 fractions to the elective lymph nodes were given, along with oral tegafur-uracil 300 mg/m^2^ every day for six weeks. Local recurrence rate was 15.5% (95% CI: 3.3–26.3). The most common late toxicity observed in these patients was bleeding from the rectal mucosa, but overall there were no unexpected serious adverse effects. The study concluded that high dose chemo-radiotherapy and watchful waiting might be a safe alternative to abdominoperineal resection in patients with locally advanced distal rectal cancer.

## CONCLUSIONS

Findings from several prospective trials have indicated that the approach of watchful waiting for patients with locally advanced distal rectal cancer who have shown complete clinical response with neoadjuvant chemotherapy and radiation might be an effective strategy. Our case series of three patients supported these findings. Our patients showed excellent response with neoadjuvant chemotherapy and radiation with a 100% disease free survival. The major limitation of our study was the small sample size and a short follow-up period. Nevertheless, it is clear from the published prospective trials that the functional results after wait-and-watch treatment are not inferior, but rather superior to outcomes after rectal surgery. In future, we plan to conduct a prospective study regarding the wait-and-watch approach for locally advanced distal rectal cancer..

## Figures and Tables

**Table 1:** Patient diagnosis, treatment regimen, and follow-up.

	Patient 1	Patient 2	Patient 3
**Age**	93	84	65
**Sex**	Female	Male	Male
**State of the disease upon diagnosis**	T3N0M0	T3N1M0	T3N1M0
**Chemotherapy and radiation regimen**	Capecitabine with radiation	Capecitabine with radiation	5FU with radiation
**Average length of follow-up**	24 months	24 months	30 months
